# Supplementary figures and images for: Rab10 regulates the sorting of internalised TrkB for retrograde axonal transport
Source: eLife. 2023 Mar 10;12:e81532. doi: 10.7554/eLife.81532 (PMC10005780; doi:10.7554/eLife.81532)

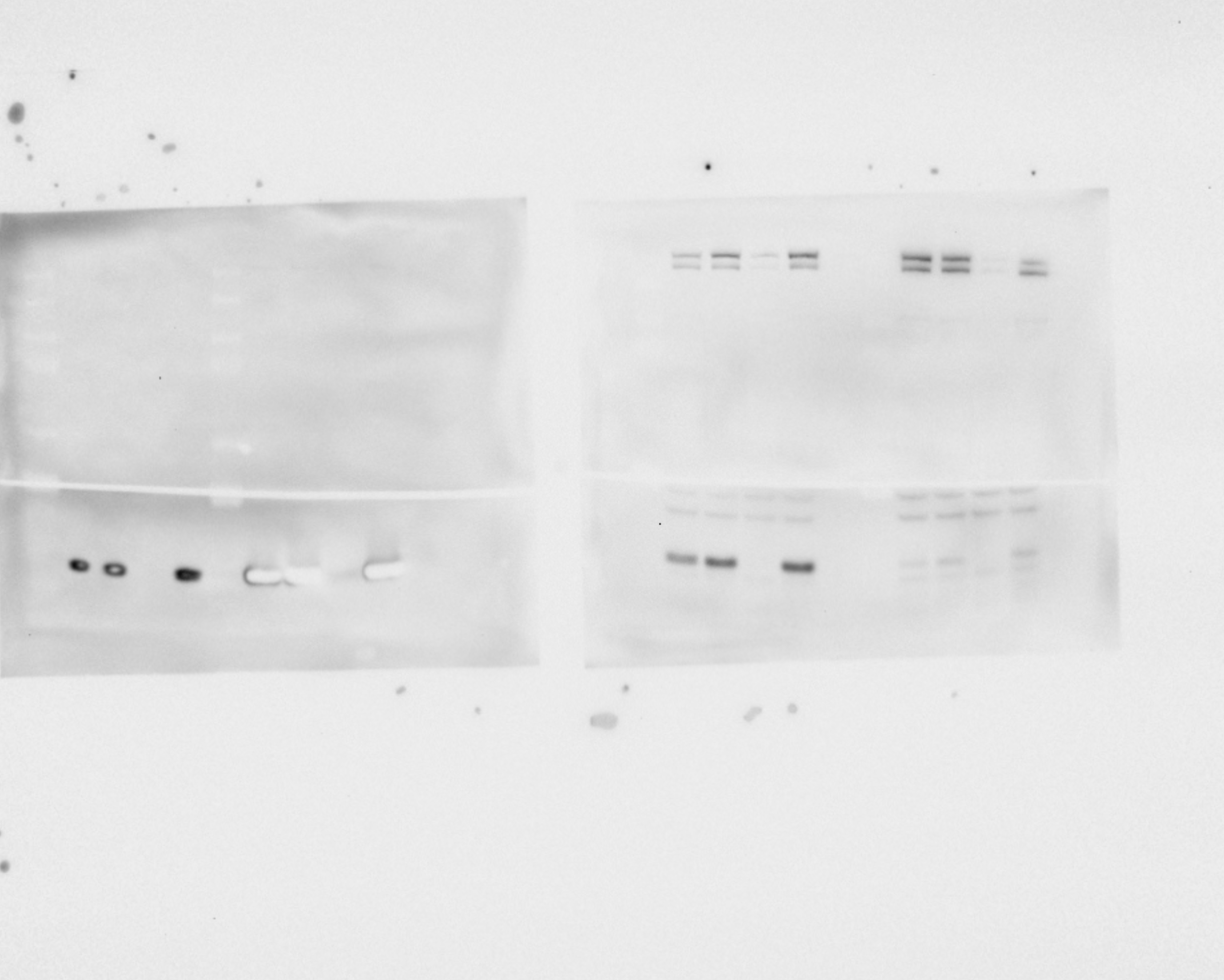

Supplement: Figure 6—source data 1. — Total cell extracts are labelled as input, and eluates from the immunoprecipitation are labelled as immunoprecipitation. Antibodies used for each membrane are indicated. Red frames indicate the bands that are shown in the main figure. [file elife-81532-fig6-data1.zip › Figure 6- source data/Figure 6g- raw image 1 (Input and IP N3).tif]

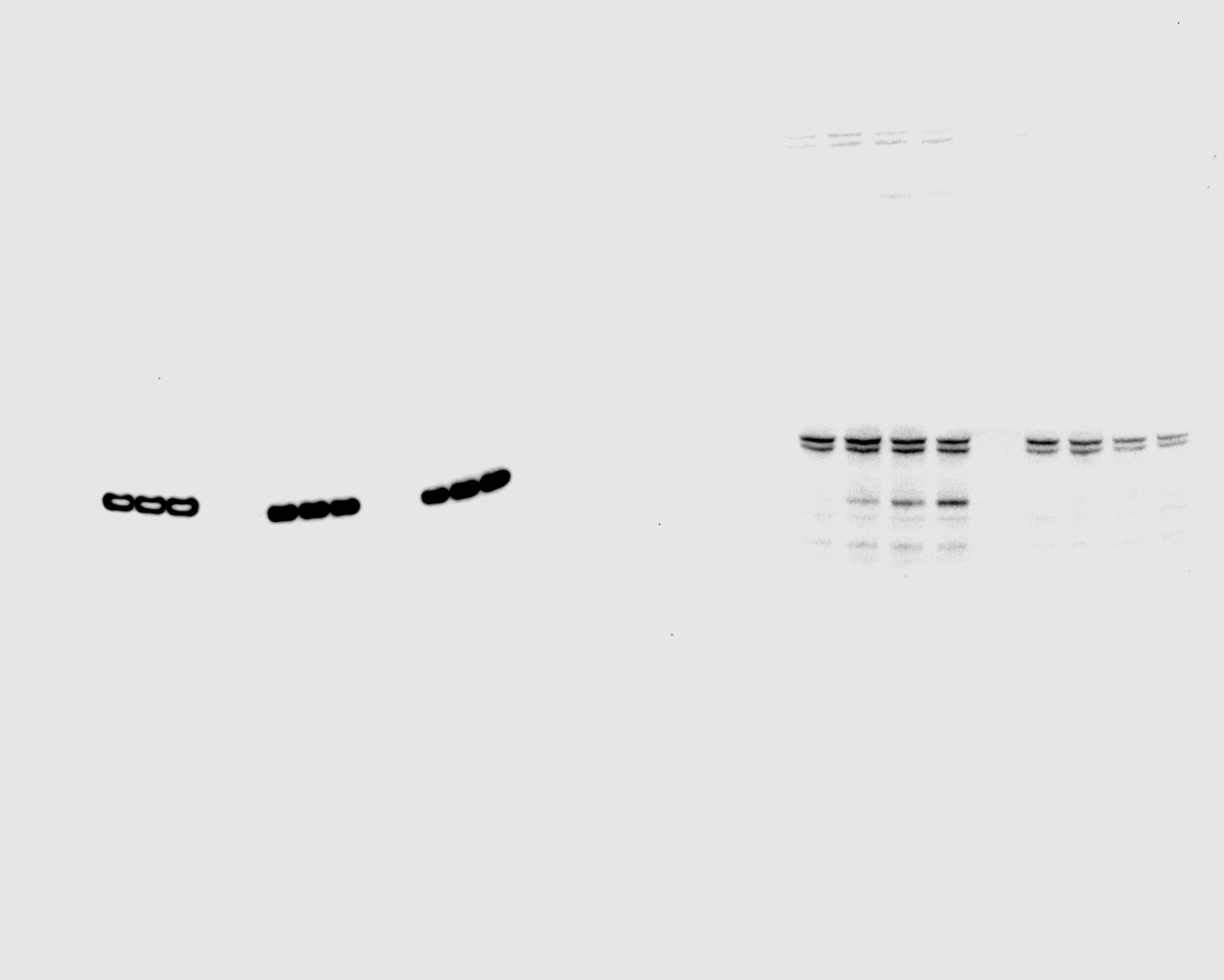

Supplement: Figure 6—source data 1. — Total cell extracts are labelled as input, and eluates from the immunoprecipitation are labelled as immunoprecipitation. Antibodies used for each membrane are indicated. Red frames indicate the bands that are shown in the main figure. [file elife-81532-fig6-data1.zip › Figure 6- source data/Figure 6g- raw image 2 (Re-blotted inputs).tif]

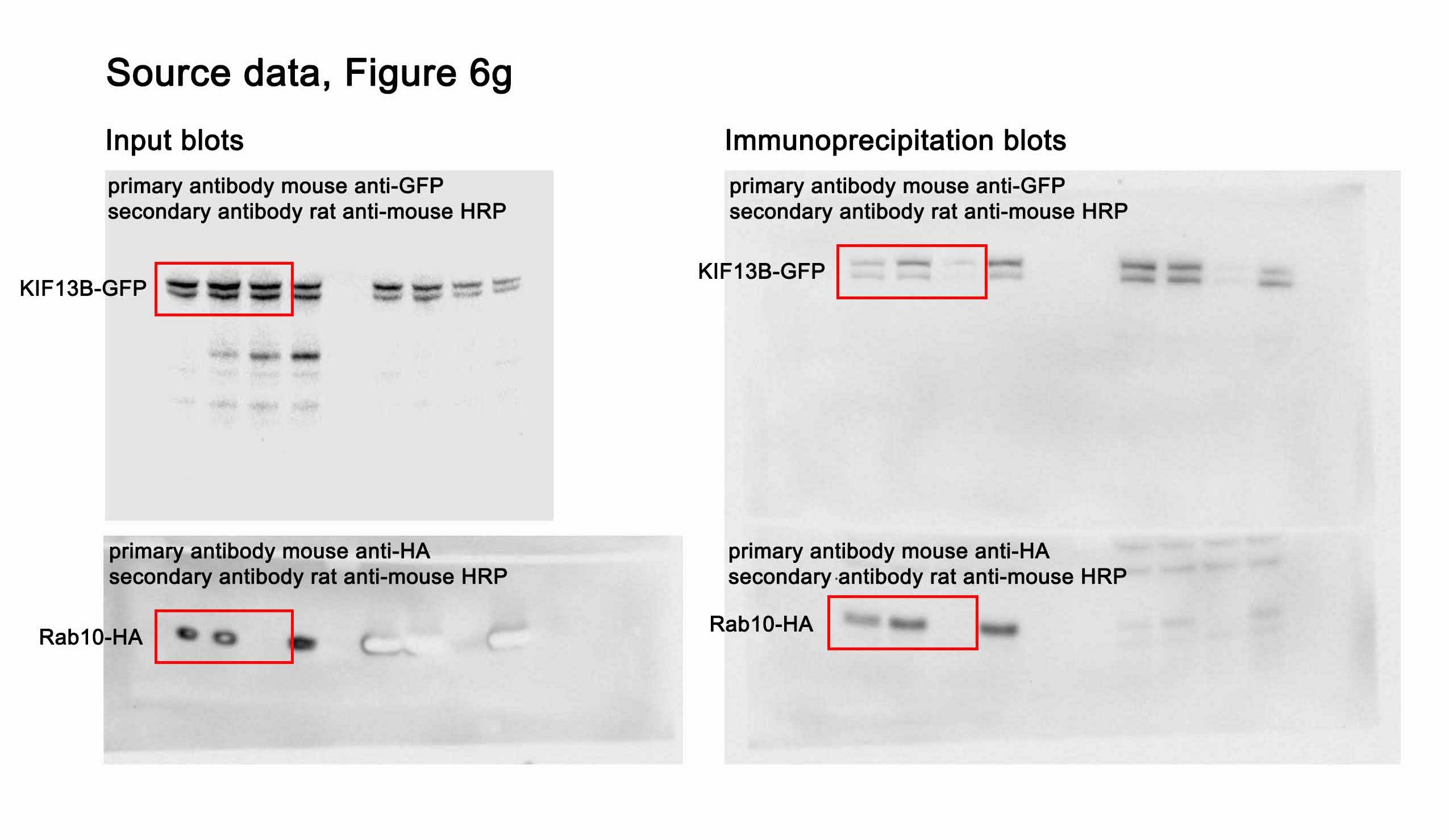

Supplement: Figure 6—source data 1. — Total cell extracts are labelled as input, and eluates from the immunoprecipitation are labelled as immunoprecipitation. Antibodies used for each membrane are indicated. Red frames indicate the bands that are shown in the main figure. [file elife-81532-fig6-data1.zip › Figure 6- source data/Figure 6g- labelled originals.jpg]
